# Colorimetric sensor array for versatile detection and discrimination of model analytes with environmental relevance

**DOI:** 10.1186/s13065-024-01181-8

**Published:** 2024-04-22

**Authors:** Mina Adampourezare, Behzad Nikzad, Sanaz Sajedi-Amin, Elaheh Rahimpour

**Affiliations:** 1 Research Center of Bioscience and Biotechnology, University of Tabriz, Tabriz, Iran; 2 Pharmaceutical Analysis Research Center and Faculty of Pharmacy, Tabriz University of Medical Sciences, Tabriz, Iran; 3 Infectious and Tropical Diseases Research Center, Tabriz University of Medical Sciences, Tabriz, Iran

**Keywords:** Colorimetric sensor array, Linear discriminant analysis, Pattern recognition, Smartphone, Determination and differentiation

## Abstract

In the current work, a rapid, simple, low-cost, and sensitive smartphone-based colorimetric sensor array coupled with pattern-recognition methods was proposed for the determination and differentiation of some organic and inorganic bases (i.e., OH^−^, CO_3_^2−^, PO_4_^3−^, NH_3_, ClO^−^, diethanolamine, triethanolamine) as model compounds. The sensing system has been designed based on color-sensitive dyes (Fuchsine, Giemsa, Thionine, and CoCl_2_) which were used as sensor elements. The color changes of a sensor array were observed by the naked eye. The color patterns were recorded using digital imaging in a three-dimensional (red, green, and blue) space and quantitatively analyzed with color calibration techniques. Distinctive colorimetric patterns for target bases via linear discriminant analysis (LDA) and hierarchical clustering analysis (HCA) were observed. The results indicated that the analytes related to each class (at the different concentration levels in the range of 0.001–1.0 mol L^−1^) were clustered together in the canonical discriminant plot and HCA dendrogram with high sensitivity and an overall precision of 85%. Furthermore, the first function factor of LDA correlated with the concentration of each target analyte in a correlation coefficient (R^2^) range of 0.864–0.996. These described procedures based on the colorimetric sensor array technique could be a promising candidate for practical applications in package technology and facile detection of pollutants.

**Supplementary Information:**

The online version contains supplementary material available at 10.1186/s13065-024-01181-8.

## Introduction

Organic and inorganic pollutants are the main classes of environmental pollution that result from the increase in the activity of refineries, fertilizers, pharmaceutical, mining, and agricultural industries [1, 2]. The inorganic pollutions include heavy metal and other inorganic pollutants such as inorganic salts, mineral bases and acids, metals, metals with organic compounds as complexes, metals compounds, cyanides, and sulfates trace elements [3]. Although inorganic pollutants exist in low concentrations in the environment, their concentration may increase with increase human activities in the industrial parts. In addition, the accumulation of organic and inorganic contaminants in the human body can lead to severe diseases and affect plants, and aquatic animals [4]. So, the detection and determination of these compounds in water, soil and even food will be valuable investigation. In the current work, hydroxide (OH^−^), carbonate (CO_3_^2−^), ammonia (NH_3_), phosphate (PO_4_^3−^), hypochlorite (ClO^−^), diethylamine (DEA), and triethylamine (TEA) were chosen as the model compounds for investigation. The chosen analytes encompass a range of chemical properties such as basicity and complexation behavior. Including these diverse analytes allows us to test the versatility of our sensor array in detecting different chemical characteristics. CO_3_^2−^ compounds are mainly obtained from the dissolution of carbonate minerals, the decomposition of organic materials, aquatic respiration, and Krebs cycle exchange. The content of these compounds can indicate the local geochemical environment [5]. NH_3_ is one of the widely used and produced chemicals in various industries. So the detection and measurement of NH_3_ in the environmental matrices is important due to its high toxicity for living cells and human health. A high NH_3_ concentration is involved in lung disorders and permanent blindness [6]. High levels of PO_4_^3−^ are one of the main reasons for the growth of harmful algae in the environment. In the presence of PO_4_^3−^, toxic algae spread uncontrollably which increase mortality of fishes and aquatic animals and reduce species diversity [7]. The ClO^−^ is added as a substance to prevent the growth of microorganisms. It delays the spoilage of food and increases its life span. This substance causes digestive problems [8]. Aqueous organic amines such as DEA, and TEA have multiple industrial applications, such as in producing insecticides, paints, rubber, and herbicides. This extensive variety of human-caused activities can have significant implications including environmental safety, human health, and water quality. For example, they can serve as precursors for the formation of different potentially hazardous substances, such as nitrosamines and nitramines. The Ministry of Ecology and Environment (GB 3838–2002) in China regards organic amines as significant water pollutants [9, 10]. Numerous methods have been employed for identifying amines in water, such as chromatographic methods (GC–MS [11], HPLC [12]) and fluorescent sensors [9, 13]. So, detecting and analyzing the concentration of organic amines and their types is crucial in aquatic environments.

So, in the past few years, increasing attention has been paid to the environmental effects of these pollutants. Although many standard methods such as ecological pollution mapping, field techniques for their measurement, and analytical determination have been developed with the aim of environmental monitoring and detection of such compounds in water, implementation of these methods is limited by experienced experts or advanced equipment [14]. One of the main limitations of using electrochemical sensors or conductive polymers is their lack of chemical selectivity, which causes the lack of differentiation of similar types [15]. Therefore, developing reliable and low-cost methods has become a high priority. Many efforts have led to the different techniques for in-situ detection of the pollutants in environmental matrices.

Colorimetric sensors are optical sensors with a low-cost and semi-quantitative property that show a detectable color change in the reaction with the analyte even with the naked eye [16]. Recently, the colorimetric sensor array has attracted attentions due to its unique features such as rapid and cost-effective analysis of multiple analytes, and ease of use [17–21]. The unique compound responses to a target analyte make this technique similar to the mammalian olfactory system. The reaction between the target analyte and the sensor elements is based on acid–base interactions, van der Waals interaction, hydrogen bonding, dipolar and multipolar interactions, and π–π molecular complexation [22]. A smartphone-based can perform a chemical analysis or medical diagnosis accurately and cost-effectively [23, 24]. Smartphone-based colorimetric sensors use reflection (not absorption) to measure color changes based on three channels: red, green, and blue (RGB). The RGB channels cover specific range of wavelengths in the red, green and blue regions of the light spectrum. The RGB color space is not directly related to the wavelength of the visible light spectrum for sensor calibration. For smartphone-based analysis to be effective, various image parameters such as hue, saturation, and value (HSV) should be considered [16, 25–27].

In the current research, we aimed to detect some organic and inorganic analytes as model compounds using a colorimetric sensor array. Color sensing materials included Fuchsine (rosaniline hydrochloride), Giemsa, Thionine, and CoCl_2._ The color changes of the sensor dyes in the presence of the target compounds were read with a smartphone equipped with a digital camera (detector).

## Materials and methods

### Materials and reagents

Sodium hydroxide, ammonium, sodium phosphate, sodium carbonate anhydrous, sodium hypochlorite, TEA, and DEA were from Merck (Germany). Fuchsine, Giemsa, thionine, and CoCl_2_ were purchased from Sigma-Aldrich (USA).

### Preparation of sensing materials to construct a colorimetric sensor array

The required stock solutions of different bases and dyes used as sensors were prepared by dissolving an appropriate amount of the desired material in ultrapure water to obtain the desired concentrations. The standard solutions of the desired analytes were prepared at concentrations of 1.0, 0.7, 0.5, 0.3, 0.1, 0.05, 0.01, and 0.001 mol L^−1^. The dyes used as sensors had the following concentrations: Fuchsine: 0.35 × 10^–3^ mol L^−1^, thionine: 0.48 × 10^–4^ mol L^−1^, CoCl_2_: 0.84 × 10^–2^ mol L^−1^, and Giemsa: 0.28 × 10^–4^ mol L^−1^.

### RGB analysis

For color analysis, sample photos were taken by a Xiaomi Poco X3 smartphone camera in the same lighting conditions, same sized area, and the same distance from the camera, and then their RGB intensity was investigated with the PhotoMetrix PRO app (the Android version).

### Colorimetric reaction and data analysis

**Fig. 1:**
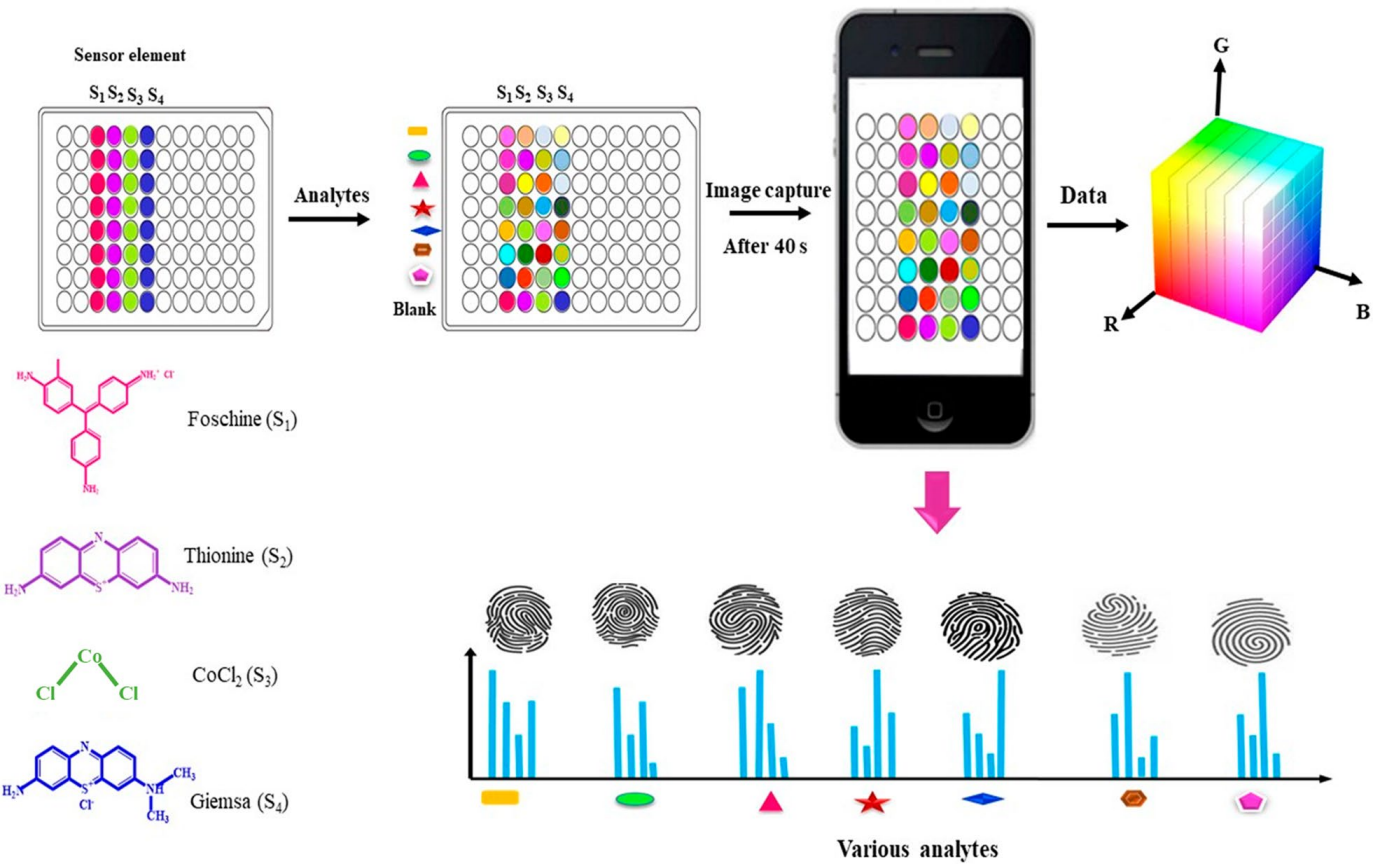
Schematic representation of the colorimetric sensing procedure and fingerprints of various bases. The image analysis using the sensor array consists of sensor elements (Fuchsine (S_1_), Thionine (S_2_), CoCl_2_ (S_3_), and Giemsa (S_4_) which are color sensitive materials, analytes (OH^−^, CO_3_^2−^, NH_3_, PO4_3_^−^, ClO^−^, DEA, and TEA), image capture device (smartphone), data acquisition (RGB values). The fingerprints of each analyte were formed by interaction between analytes and can be distinguished due to the distinctive color map profiles

During the reaction steps, 150 μL of each base with different concentrations were reacted with 150 μL of dyes. Each sensing material was added to the corresponding well using a multi-channel micropipette. The color changes of the reaction were photographed at different times of 15 s, 20 s, 25 s, 30 s, 40 s, 50 s, 60 s, 2 min, 3 min, 5 min, and 7 min using a smartphone. For this purpose, a handcrafted rectangular photography box containing two LED lamps on both sides of the box was designed. The reaction plate was placed inside the box to provide adequate lighting for image acquisition, and photography was done using a smartphone camera using a hole drilled into the top of the box. All reactions were performed at room temperature. Seven bases and four color sensors made up this sensor array (Fig. 1). The reactions were performed on a 96-well plate. A blank experiment was also performed in the same manner using ultrapure water instead of the analyte solution. After digital data acquisition, the average color values (R: red, G: green, and B: blue) were extracted from the center of each sensor spot in the sensor array images collected multiple times. The changes in RGB color values (ΔR, ΔG, and ΔB) were calculated using Eqs. (2)–(4).

1 $$ {\text{EDs}} = \left( {\left( {{\text{R}}{-}{\text{b}}} \right)^{{2}} + \left( {{\text{G}}{-}{\text{b}}} \right)^{{2}} + \left( {{\text{B}}{-}{\text{b}}} \right)^{{2}} } \right)^{{{1}/{2}}} $$

2 $$ \Delta {\text{R}} = \left| {\left( {{\text{R}}_{{{\text{AF}}}} {-}{\text{R}}_{{{\text{BF}}}} } \right) - \left( {{\text{R}}_{{{\text{AI}}}} - {\text{R}}_{{{\text{BI}}}} } \right)} \right| $$

3 $$ \Delta {\text{G}} = \left| {\left( {{\text{G}}_{{{\text{AF}}}} {-}{\text{G}}_{{{\text{BF}}}} } \right) - \left( {{\text{G}}_{{{\text{AI}}}} - {\text{G}}_{{{\text{BI}}}} } \right)} \right| $$

4 $$ \Delta {\text{B}} = \left| {\left( {{\text{B}}_{{{\text{AF}}}} {-}{\text{B}}_{{{\text{BF}}}} } \right){-}\left( {{\text{B}}_{{{\text{AI}}}} {-}{\text{B}}_{{{\text{BI}}}} } \right)} \right| $$

The subscripts A and B show the sample and blank, and I and F show the initial and final images, respectively. The total squared Euclidean distances (EDs, i.e., the sum of the squares of each ΔRGB color value of the four sensing materials) were then calculated. For visualization, color difference images were generated using averaged absolute RGB color change values by Microsoft PowerPoint, version 2013. The spots of sensing elements were illustrated in an image after adding studied analytes in Additional file 1: Fig. S1. IBM SPSS 22.0 software was used for LDA and HCA analysis. The statistical parameters were also computed with MATLAB 2014 and classification toolbox 5.4. The validation of the model was carried out by calculating the sensitivity (True-positive (TP) / TP + False-negative), specificity (True-negative (TN)/TN + False-positive), and accuracy (TP + TN/sample size).

## Results and discussion

### Principle of the colorimetric sensor array

The most important part of the sensor array is choosing sensor elements. Sensor array is a multidimensional platform with improved sensitivity and modified discrimination ability. The colorimetric sensor array was designed by considering two requirements: (1) sensors react with target analytes, and (2) the reaction between the sensors and the analytes is accompanied by a color change. The color sensitive materials used in this study were Fuchsine (rosaniline hydrochloride), Giemsa, Thionine, and CoCl_2_. These species have chromogenic groups and are capable of color change.

The colorimetric responses of Fuchsine, a triarylmethane dye, unveil its dynamic behavior when exposed to various chemical agents. The principle underlying these transformations lies in the susceptibility of fuchsine's chemical structure to interactions with specific reagents. In the presence of OH^−^, ClO^−^, DEA, and TEA, Fuchsine undergoes notable degradation with a discernible shift from original vivid pink to a pale gradient pink shade. The interaction with OH^−^ suggests potential alterations in Fuchsine's chemical composition, possibly involving changes in its molecular structure. Furthermore, the responsiveness of fuchsine to ClO^−^, DEA, and TEA underscores its versatility as a sensor element within the array method. The observed color changes reflect nuanced reactions to a spectrum of chemical stimuli, allowing for a comprehensive analysis of diverse analytes [28–30]. In the sensor array method, the sensor elements respond not only to target analytes but also to others to varying degrees, as we do not have specific sensors but rather a variety of cross-reactive sensors.

Thionine, a cationic thiazine dye employed as a sensor element, holds promise for charge transfer (CT) interactions in our colorimetric system. In the presence of amines, previous studies have identified thionine's tendency for involving in CT interactions. Thionine, as a cationic thiazine dye, generally acts as an electron acceptor in CT reactions. In interactions with amines, thionine tends to accept electrons from the amine molecules and form a thionine-amine CT complex. The general representation of this reaction can be expressed as follows:

Thionine^+^  + Amine ⟶Thionine-Amine CT Complex

This characteristic behavior contributes to the observed color changes in the sensor array when interacting with various analytes [31, 32].

The diverse color changes of CoCl_2_ make it an excellent candidate for colorimetric-based reactions, offering a visually accessible means to detect and quantify the presence of specific analytes or the occurrence of chemical reactions. The chemical behavior of CoCl_2_ varies according to conditions, involving processes like complexation reactions, redox reactions, precipitation reactions, displacement reactions, and substitution reactions. The aqueous solution of CoCl_2_ is pink, while this color turns blue in reaction with OH^−^ and CO_3_^2−^ ions. This reaction is based on the displacement reaction. Also, the color change provided by the amines-CoCl_2_ reaction is due to the complex formation. ClO^−^ ion is a potent oxidizing agent. By adding sodium hypochlorite to CoCl_2_ aqueous solution, the color changes to a dark brown precipitate, which is probably due to the oxidation of Co (II) [33, 34].

The Giemsa stain, a pivotal color-sensitive component in this study, is a complex dye mixture comprising azure B, methylene blue, and eosin. This sophisticated composition imparts versatility to the Giemsa stain as it can undergo distinctive color changes based on the surrounding conditions. Typically, Giemsa stain solutions appear to be a deep purple or violet color. When applied to an analyte, the stain may produce shades of purple or blue-purple. Precisely, under excessively basic conditions, the color of the Giemsa shifts towards blue, highlighting its sensitivity to changes in pH. The blue color of Giemsa stain under basic conditions (such as at pH 12) is attributed to the presence of methylene blue, one of the components in the Giemsa stain mixture. Conversely, in high acidic conditions (pH = 1–2), the stain exhibits a red hue due to the presence of the eosin compound within the mixture. This dual-color responsiveness adds a nuanced layer to the sensor array's capabilities, allowing for a broader range of analyte interactions to be discerned [35, 36]. Therefore, any color change due to Giemsa-analyte interactions can be interpreted accordingly.

### Optimization of the colorimetric sensor array

To evaluate the efficiency of the proposed array sensor, it was necessary first to examine the effect of parameters in creating a distinct color pattern. One of the essential parameters was the concentration of chemo-sensitive dye. The concentration of these sensor elements was optimal when it can exhibit the lowest concentration of our analytes with detectable changes. The optimum concentration of dyes used as sensors had the following concentration: Fuchsine: 0.35 × 10^–3^ mol L^−1^, thionine: 0.48 × 10^–4^ mol L^−1^, CoCl_2_: 0.84 × 10^–2^ mol L^−1^, and Giemsa: 0.28 × 10^–4^ mol L^−1^.

**Fig. 2:**
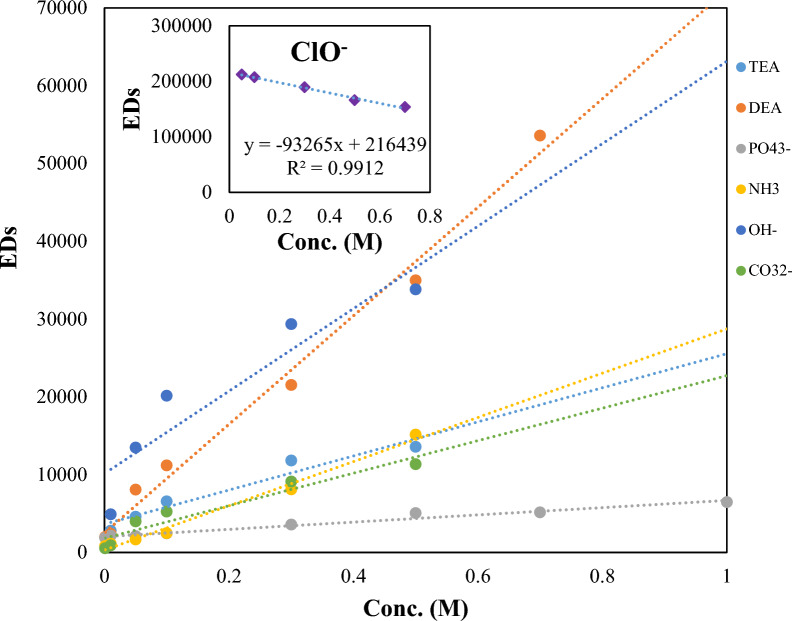
Total EDs versus concentration curves for each organic compound at the concentration range of 0.001–1.0 mol L^−1^ Feasibility of quantitative analysis using sensor array. Overall sensor response for each analytes (i.e., ED from the changes in all RGB values from 4 sensor elements) versus concentration were shown

To determine the optimum reaction time, we have investigated the kinetics of each reaction for sensor array-based analysis. The sensor's response time needs to be optimized since it needs to be adequate (that is, not too short to create unreliable results or too long to prolong our analysis time). Since the reaction time depends on the concentration of the analytes, the regression coefficient of bases, as a time-dependent parameter, was also optimized. The regression coefficients from the linear regression of the total square Euclidean distance with the concentration at different time points up to 7 min were compared (Fig. 2 and Additional file 1: Fig. S2). Most of the results show a gradual decrease in the regression coefficients. However, in some cases, the regression coefficient has remained constant. So, achieving an optimum reaction time for the proposed sensor array involves the shortest time with a regression coefficient above 0.9. Based on the results, a reaction time of 40 s was chosen for subsequent experiments. The linearity range for all kinds of bases was as follows: OH^−^ (0.01–0.5 mol L^−1^, ≥ 0.94), CO_3_^2−^ (0.001–0.5 mol L^−1^, ≥ 0.91), PO_4_^3−^ (0.001–1.0 mol L^−1^, ≥ 0.97), NH_3_ (0.001–0.5 mol L^−1^, ≥ 0.98), ClO^−^ (0.05–0.7 mol L^−1^, ≥ 0.99), DEA (0.001–0.7 mol L^−1^, ≥ 0.98), TEA (0.01–0.5 mol L^−1^, ≥ 0.93). These results suggest that the validated colorimetric sensor array could be applied to both semi-quantitative and qualitative detection of studied bases without need for a time-consuming analytical process.

**Fig. 3:**
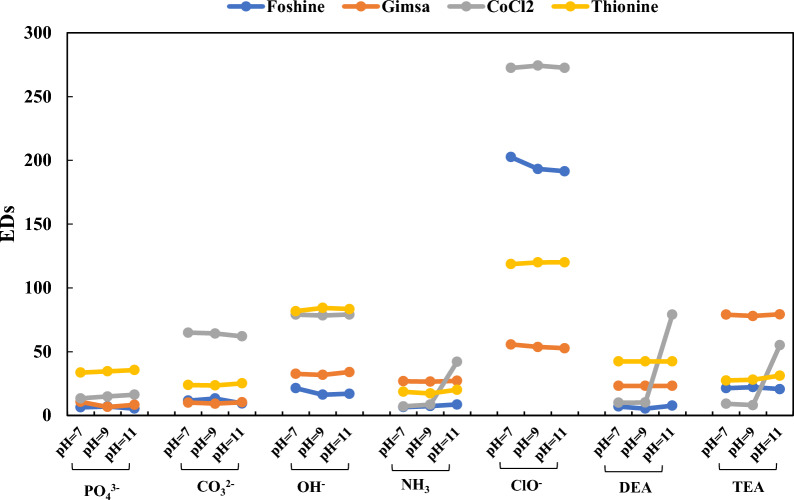
The pH responses of the total EDs of each organic compound (0.1 M) in different sensor elements. The experiments at three pH levels (7, 9, and 11) were conducted and assess the potential pH sensitivity of these pigments

In our colorimetric sensor array, we investigated the response of pigments, including Fuchsine (rosaniline hydrochloride), Thionine, Gimsa, and CoCl_2_, to varying pH levels in the presence of studied analytes. We designed our experiments to explore the potential pH sensitivity of these pigments and assess their performance in a range of pH conditions. Specifically, we conducted experiments at three pH levels (7, 9, and 11), and our results indicated that CoCl_2_ exhibited a noticeable color change in the presence of analytes. However, the other pigments, namely Fuchsine, Thionine, and Gimsa, showed minimal or no discernible color changes across the tested pH range. The lack of significant color changes in these pigments across different pH levels (Fig. 3), indicated that the sensing actions were resulted from the reaction between color dyes and ions and bases. Also, to overcome the concerns regarding the color change of CoCl_2_ we checked the pH value of NH_3_, DEA, and TEA solutions with different concentrations in our range of study. The pH values change in 0.5 units which doesn’t make significant changes.

### Colorimetric sensor responses

**Fig. 4:**
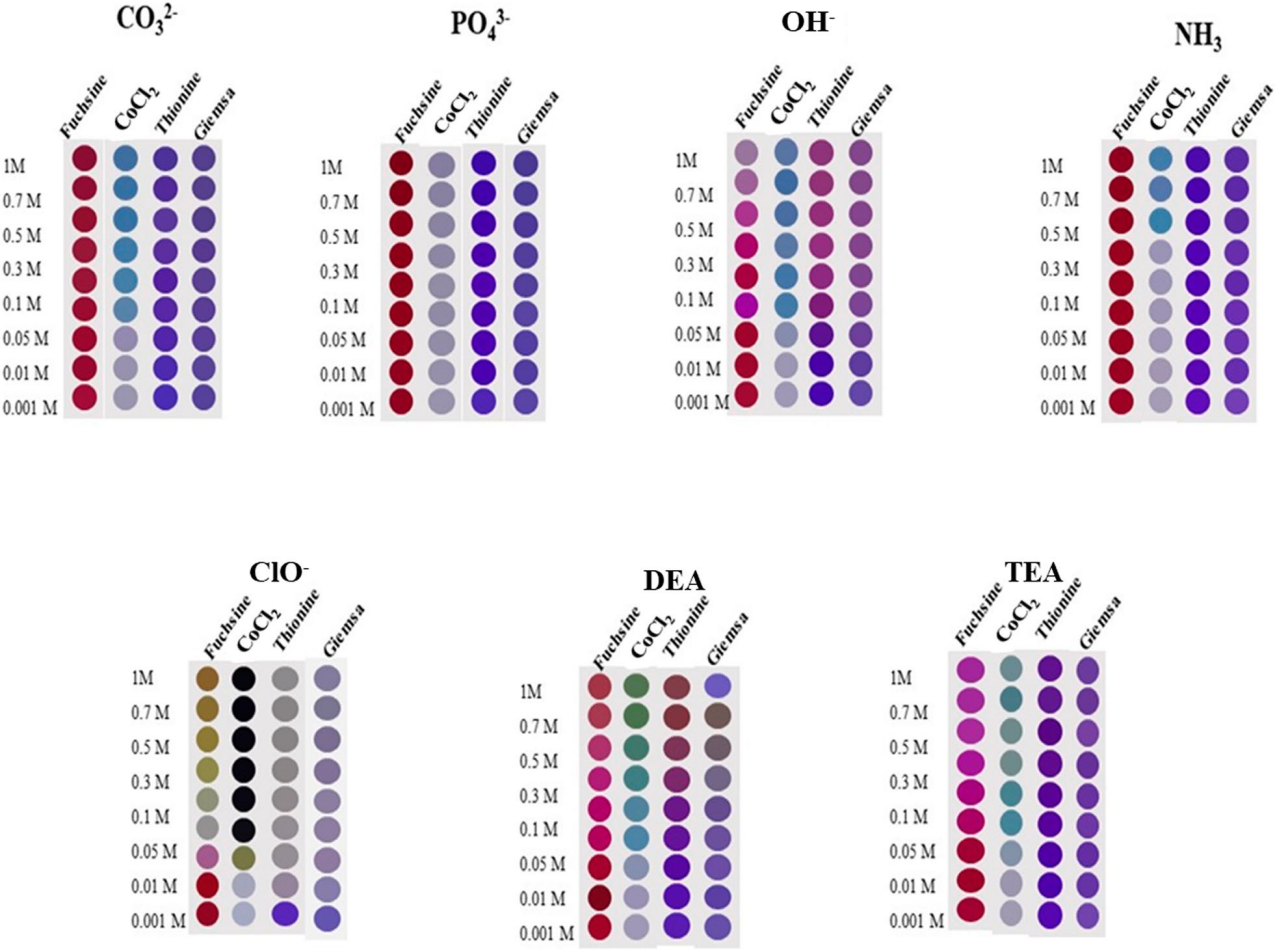
The difference image maps obtained for various bases at different concentrations. The RGB color difference (ΔRGB = RGB_BI_ − RGB_AF_) for various bases at different concentrations was calculated and the spot colors were constructed according to their calculated values in Microsoft PowerPoint

To illustrate the use of a colorimetric sensor array for bases discrimination, the difference image maps obtained for the seven bases at eight concentrations were shown in Fig. 4. Different bases were prepared at a range of concentrations (1.0, 0.7, 0.5, 0.3, 0.1, 0.05, 0.01, and 0.001 mol L^−1^). This range was chosen to cover a broad range of possible concentrations encountered in real-world samples. By doing so, the accuracy and reliability of the determination method for various concentrations of studied analytes are ensured.

The cross-responsive interaction between analytes and the sensor array generates specific patterns like fingerprints for each base. According to Fig. 4, the different bases can be distinguished due to the distinctive color map profiles. More specifically, the color maps obtained for OH^−^, ClO^−^, and DEA were considerably different from the other bases. Therefore, it can be predicted that the bases mentioned above can be detected with high sensitivity. Also, by comparing the color pattern for different concentrations of bases, it can be seen that the higher the concentration, the greater the color changes.

### Colorimetric Pattern Recognition with HCA

**Fig. 5:**
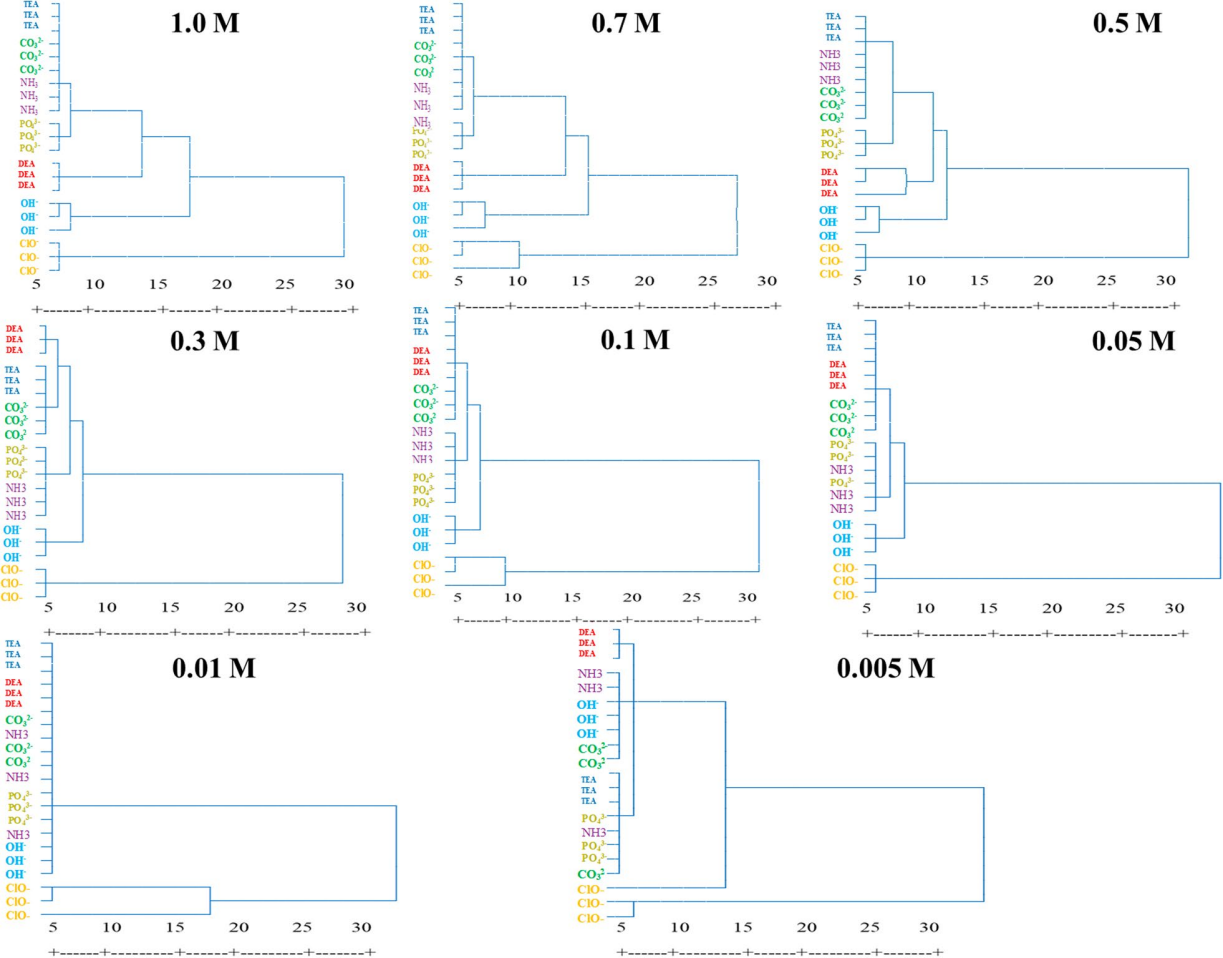
The HCA results among seven investigated bases with concentrations 1.0, 0.7, 0.5, 0.3, 0.1, 0.05, 0.01, and 0.001 mol L^−1^. The colorimetric response for given concentrations (4 sensing elements × 3 colorimetric channels × 7 type of bases × 3 replicates) were analyzed by HCA in IBM SPSS 22.0 software and results were shown in this figure. The dendrograms, which had the most similar color patterns, were grouped together

After observing the specific difference image maps, we employed statistical multivariate analysis methods to demonstrate the RGB variations more clearly. So, the ability of the sensor array to discriminate OH^−^, CO_3_^2−^, PO_4_^3−^, NH_3_, ClO^−^, DEA, and TEA was evaluated by HCA methods. HCA, as an unsupervised clustering algorithm, provides discrimination of different analytes. These methods are widely used for visualization and identifying patterns in multidimensional data. For this purpose, different concentrations of bases were added to each sensor, and the colorimetric response (RGB color value) was obtained before and after adding the bases. The changes in RGB color values (ΔR, ΔG, and ΔB) were used to construct the colorimetric response patterns for bases at different concentrations. Thus, for a given concentration, the raw data matrix (4 sensing elements × 3 colorimetric channels × 7 type of bases × 3 replicates) was obtained. Figure 5, showed the individual HCA dendrograms among seven investigated bases with concentrations of 1.0, 0.7, 0.5, 0.3, 0.1, 0.05, 0.01, and 0.001 mol L^−1^. In each dendrogram, analytes were successively clustered in the arrangement of their likenesses in a 12-dimensional vector space. These results revealed that the sensor array successfully identified the seven bases at concentrations 1.0–0.1 mol L^−1^. However, the lower concentrations of bases were not well separated and were incorrectly grouped in the HCA. In most of the dendrograms, the observations related to NH_3_, CO_3_^2−^, PO_4_^3−^, and TEA were grouped which showed that they had the most similar color patterns. On the other hand, OH^−^ and ClO^−^ were often clustered in a separate category, which showed that they had distinct color patterns from other bases.

### Colorimetric pattern recognition with LDA

The multidimensional response pattern generated by the sensor array in the presence of the seven bases was also analyzed by LDA. The values related to the eigenvalues and the contribution of each factor in describing the data variance for different concentrations of bases were calculated. The results showed that for all eight different concentrations of bases, the first two factors had described 100% of the total variance.

**Fig. 6:**
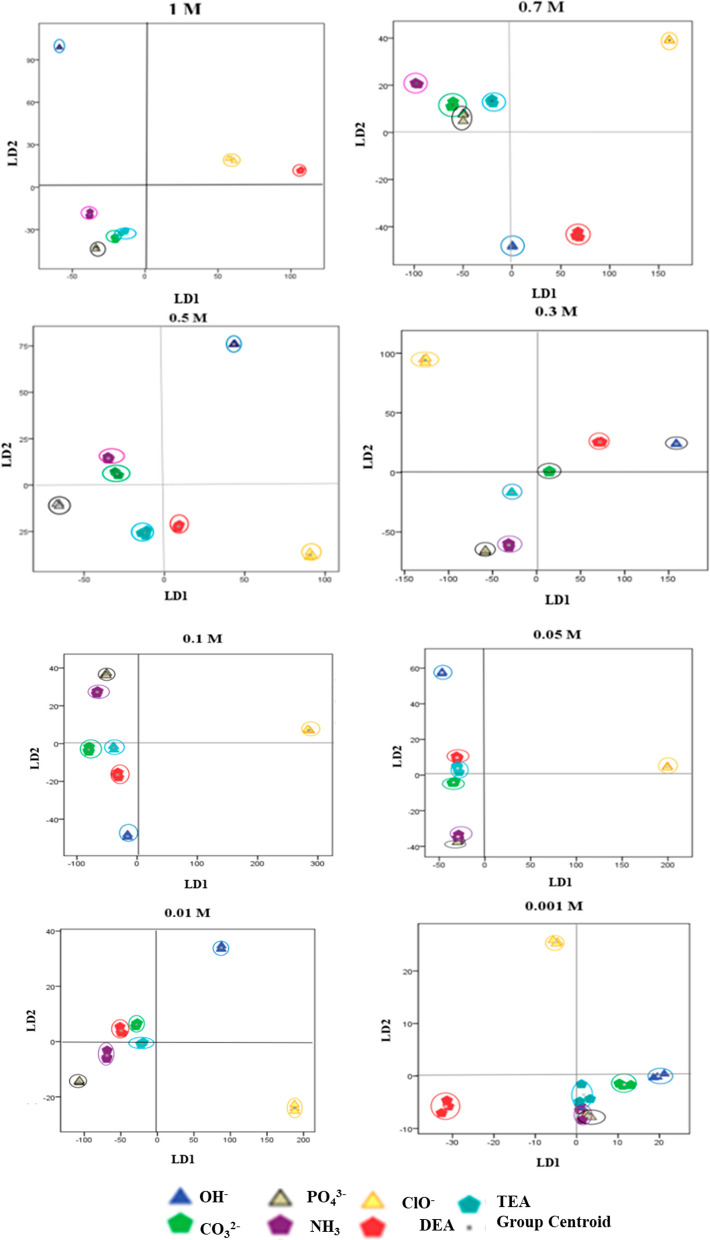
The LDA results among seven investigated bases with concentrations 1.0, 0.7, 0.5, 0.3, 0.1, 0.05, 0.01, and 0.001 mol L^−1^. The colorimetric response for given concentrations (4 sensing elements × 3 colorimetric channels × 7 type of bases × 3 replicates) were analyzed by LDA in IBM SPSS 22.0 software and results were shown in this figure. No overlap, which indicated that the bases could be discriminated in concentrations

The results of LDA (Fig. 6) showed that the colorimetric response pattern for different concentrations of each base was significantly classified so that each group belonged to a specific base. In addition, the distance between the groups indicated the optimal discrimination capability of the proposed array sensor. In contrast, the distance between repeated measurements of each analyte has a very narrow distribution. In other words, the LDA method has been able to distinguish and visualize the color patterns created by different bases by reducing the data dimensions. The results obtained from LDA and HCA are consistent. As illustrated in Fig. 5 (HCA results), the considerable between group distance observed for OH^−^ and ClO^−^ compared to other analyte groups in LDA plots indicated a distinct pattern discerned by our sensor array for these specific classes. Conversely, the NH_3_, PO_4_^3−^ and CO_3_^2−^ groups consistently displayed a minimal distance between their group centroids, suggesting similar responses. From the LDA plots, we could see the discrimination ability of the proposed sensor array in the concentration range of 0.1–1 mol L^−1^ of each base. However, in LDA plots of bases in the concentration range of 0.001–0.05 mol L^−1^, severe overlap existed (except OH^−^ and ClO^−^), which indicated that the bases could not be discriminated in concentrations below 0.05 mol L^−1^. To test the accuracy of the prediction of a new sample group with the LDA method, the cross-validation method was used. Table 1 indicated the results of classification by LDA and cross-validation using the proposed colorimetric sensor array for seven bases. The error rate of the LDA model was 0.14 (19%), 0.00, 0.10, 0.10, 0.00, 0.43, 0.05, and 0.38 in cross-validation for eight concentration levels of studied inorganic compounds. The performance of the model was evaluated, and detailed classification results were also shown in Table 1. The overall specificity, sensitivity, and precision values of the LDA model were 85%, 98%, and 85%, respectively. These results indicate that the proposed colorimetric sensor array could recognize the difference in base profiles and discriminate them with some degree of accuracy.

**Table 1:** The classification results of the LDA model for different concentrations of bases

	OH^−^	CO_3_^2−^	PO_4_^3−^	NH_3_	ClO^−^	DEA	TEA
*1.0 mol L*^*−1*^							
Sensitivity	1	0.67	0.67	0.67	1	1	1
Specificity	1	0.94	1	1	1	1	0.89
Precision	1	0.67	1	1	1	1	0.6
*0.7 mol L*^*−1*^							
Sensitivity	1	1	1	1	1	1	1
Specificity	1	1	1	1	1	1	1
Precision	1	1	1	1	1	1	1
*0.5 mol L*^*−1*^							
Sensitivity	1	1	1	0.33	1	1	1
Specificity	1	0.94	0.94	1	1	1	1
Precision	1	0.75	0.75	1	1	1	1
*0.3 mol L*^*−1*^							
Sensitivity	0.67	0.67	1	1	1	1	1
Specificity	1	1	1	1	1	0.89	1
Precision	1	1	1	1	1	0.6	1
*0.1 mol L*^*−1*^							
Sensitivity	1	1	1	1	1	1	1
Specificity	1	1	1	1	1	1	1
Precision	1	1	1	1	1	1	1
*0.05 mol L*^*−1*^							
Sensitivity	0.67	0	0	0.33	1	1	1
Specificity	1	1	0.89	0.78	1	0.89	0.94
Precision	1	0	0	0.2	1	0.6	0.75
*0.01 mol L*^*−1*^							
Sensitivity	1	1	1	0.67	1	1	1
Specificity	1	1	1	1	1	1	0.94
Precision	1	1	1	1	1	1	0.75
*0.001 mol L*^*−1*^							
Sensitivity	0.67	0	0.33	0.67	0.67	1	1
Specificity	0.94	0.89	0.94	0.89	1	1	0.89
Precision	0.67	0	0.5	0.5	1	1	0.6

In order to further investigate the application of the sensor array to real samples, it was used to identify studied analytes in river water sample obtained from Tabriz, Iran. For this purpose, the river water samples were diluted 50-fold with water and were separately spiked with 0.1 mol L^−1^ of each analyte. The LDA results were shown in Additional file 1: Fig. S3. The river water sample alone (used as a control) produced a distinct array response, allowing for the differentiation of the seven individual analytes. The first two conventional factors included 60.9 and 38.5% of variance, which accounted for more than 99% of the total variance.

### Discrimination of different bases at various concentrations

**Fig. 7:**
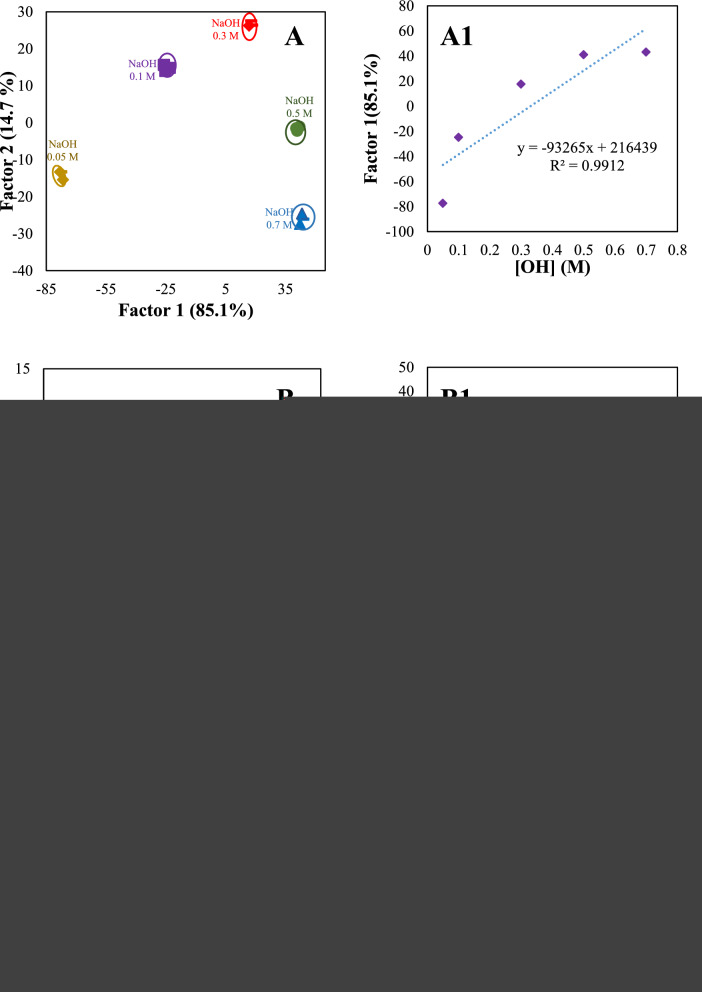

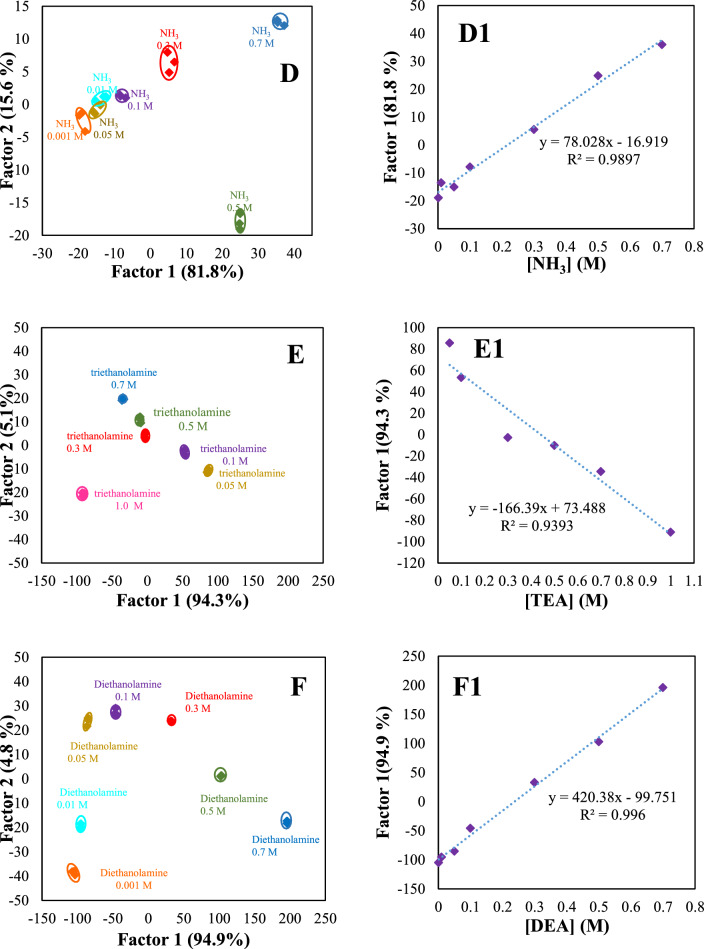

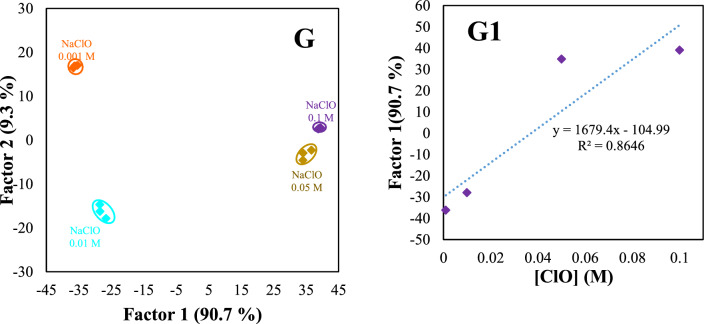
Canonical discriminant function and linearity range for OH^−^ (**A**, **A1**), CO_3_^2−^ (**B**, **B1**), PO_4_^3−^(**C**, **C1**), NH_3_ (**D**, **D1**), TEA (**E**, **E1**), DEA (**F**, **F1**), and ClO^−^ (**G**, **G1**) at different concentrations. The discrimination ability of sensor arrays for the identification of analytes at various concentrations is shown (**A**, **B**, …, **G**). The quantitative range of the proposed sensor array for each bases were shown in **A**_1_, **B**_1_, …, **G**_1_

The discrimination ability of sensor arrays for the identification of analytes at various concentrations is commonly regarded as challenging. The discrimination pattern for each analyte at multiple concentrations (0.001, 0.01, 0.05, 0.1, 0.3, 0.5, 0.7, and 1.0 mol L^−1^) was shown in Fig. 7. The sensitive range of the proposed sensor array was obtained from 0.05 to 0.7 mol L^−1^ for OH^−^ (Fig. 7A, A1), 0.05−0.5 mol L^−1^ for CO_3_^2−^ (Fig. 7B, B1), 0.01−1.0 mol L^−1^ for PO_4_^3−^ (Fig. 7C, C1), 0.001−0.7 mol L^−1^ for NH_3_ (Fig. 7D, D1), 0.05−1.0 mol L^−1^ for TEA (Fig. 7E, E1), and 0.001−0.7 mol L^−1^ for DEA (Fig. 7F, F1), and 0.001−0.1 mol L^−1^ for ClO^−^ (Fig. 7G, G1).

Figure 7A1–G1 shows an excellent linear relationship between discriminant functions and the concentration of studied analytes with a strong correlation coefficient in the range of (R^2^ = 0.86–0.996). The results indicate that our sensor array has the potential to serve as an initial screening tool for measuring the levels of the targeted analytes. This screening capability allows for efficient detection of our studied analytes. However, traditional analytical techniques such as chromatography are still necessary for more precise measurements. This suggests that the sensor can provide a preliminary assessment of the analyte amounts but should be complemented with more accurate methods for more detailed analysis.

### Interference effect

**Fig. 8:**
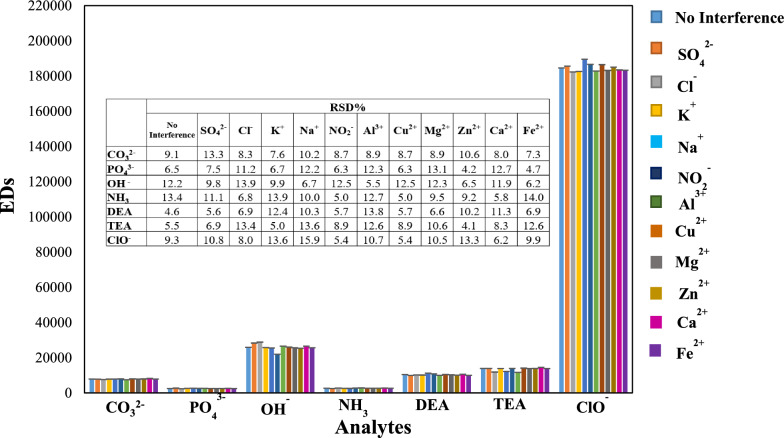
Evaluation of the repeatability of the developed sensor array to studied analytes (blue bar chart, No Interference) and considering some coexisting ions as common interference. The repeatability assessment of our developed sensor array with their relative standard deviation (RSD%)

The effect of interference on the efficiency of our developed sensor array for detecting the studied analytes has been evaluated. For this purpose, some coexisting ions such as SO_4_^2−^, NO_2_^−^, Cl^−^, K^+^, Al^3+^, Cu^2+^, Mg^2+^, Zn^2+^, Ca^2+^, Fe^2+^ and Na^+^ were used. The color pattern obtained from the developed sensor array for each analyte with a concentration of 0.1 mol L^−1^ in the presence of various aforementioned interferents with a concentration of 1 mol L^−1^ were analyzed. The color change of the sensor array was demonstrated as EDs in Fig. 8. As can be seen, the presence of the mentioned interferences did not change the color pattern of the analytes.

### Repeatability of method

In order to repeatability assessment of the developed sensor array, the response toward the investigated bases was analyzed (seven individual experiments where each one was replicated three times). The relative standard deviation (RSD) for each analyte was obtained and mentioned in Fig. 8. The results showed that the value of the RSD % was less than 14%. This suggested that the repeatability of our sensor array was acceptable for semiquantitative analyses.

## Conclusion

In this work, a simple and rapid smartphone-based colorimetric sensor array was developed for the detection and discrimination of some organic and inorganic bases as a case study. The sensing technique offers a series of unspecific interactions between the analytes and the sensor elements, leading to various color changes. Smartphone as digital image analyzer utilizes triple-channels (RGB) coupled with multivariate analysis for pattern recognition. The LDA and HCA results could discriminate seven investigated bases (OH^−^, CO_3_^2−^, PO_4_^3−^, NH_3_, ClO^−^, DEA, TEA) at different levels of concentrations. Furthermore, the sensor array could efficiently differentiate the individual analytes in their sensitive range of concentrations.

### Supplementary Information

**Additional file 1: Fig. S1.** Representative final colorimetric sensor array image for a studied analytes (0.3 M) under optimum conditions. The rows represent the type of analyte and the columns represent the type of our sensor element. **Fig. S2.** The color response linearity at different reaction times. **Fig. S3.** LDA results upon the addition of studied analytes to river water samples.

## Data Availability

The datasets used and/or analysed during the current study are available from the corresponding author on reasonable request.
