# Supplementary material for: Colorimetric sensor array for versatile detection and discrimination of model analytes with environmental relevance
Source: BMC Chem. 2024 Apr 22;18(1):80. doi: 10.1186/s13065-024-01181-8 (PMC11034120; doi:10.1186/s13065-024-01181-8)
Supplement: Supplementary file 1 — Additional file 1: Fig. S1. Representative final colorimetric sensor array image for a studied analytes (0.3 M) under optimum conditions. The rows represent the type of analyte and the columns represent the type of our sensor element. Fig. S2. The color response linearity at different reaction times. Fig. S3. LDA results upon the addition of studied analytes to river water samples. [file 13065_2024_1181_MOESM1_ESM.docx]

**"Additional Information"**

**Colorimetric Sensor Array for Versatile Detection and Discrimination of Model Analytes with Environmental Relevance**

Mina Adampourezare ^a,b^, Behzad Nikzad ^a^, Sanaz Sajedi-Amin ^b,*^, Elaheh Rahimpour ^b,c,*^

**^a^** *Research Center of Bioscience and Biotechnology, University of Tabriz, Tabriz, Iran*

*^b^ Pharmaceutical Analysis Research Center and Faculty of Pharmacy, Tabriz University of Medical Sciences, Tabriz, Iran*

*^c^ Infectious and Tropical Diseases Research Center, Tabriz University of Medical Sciences, Tabriz, Iran*

*Corresponding authors E-mail addresses: [rahimpour_e@yahoo.com](mailto:rahimpour_e@yahoo.com), [sv.sajedi@gmail.com](mailto:sv.sajedi@gmail.com)

Sanaz Sajedi-Amin is co-corresponding author in this work.

| 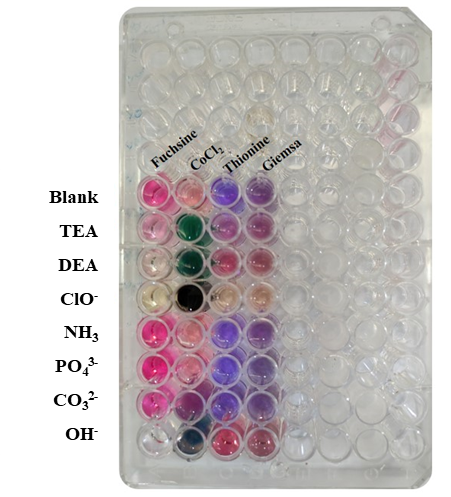 |
| --- |
| **Fig. S1.** Representative final colorimetric sensor array image for a studied analytes (0.3 M) under optimum conditions. The rows represent the type of analyte and the columns represent the type of our sensor element. |

|  |
| --- |
| **Fig. S2.** The color response linearity at different reaction times. |

| 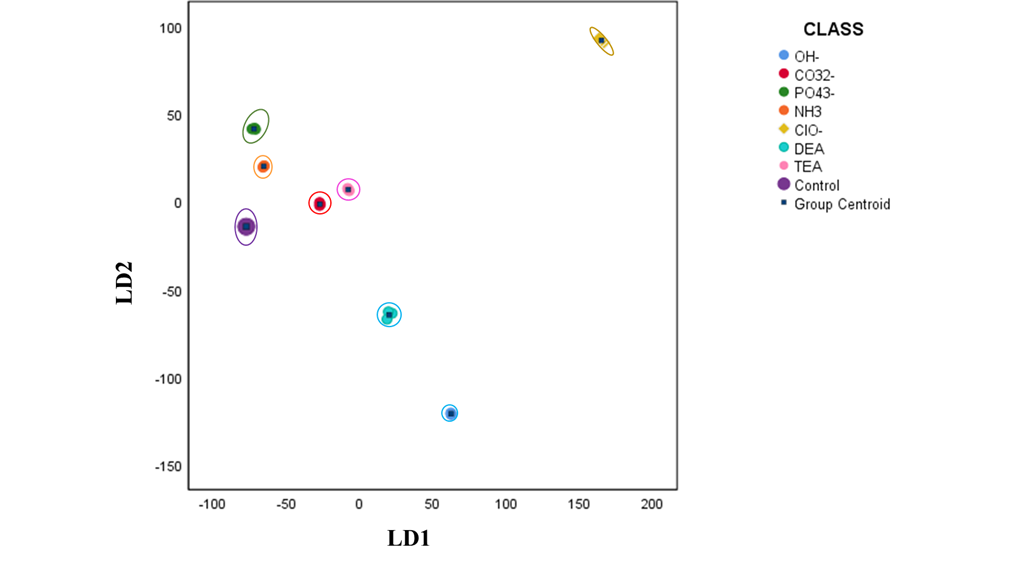 |
| --- |
| **Fig. S3.** LDA results upon the addition of studied analytes to river water samples. |
